# The Incidence of Cardiovascular Events Is Comparable Between Normoalbuminuric and Albuminuric Diabetic Patients With Chronic Kidney Disease

**DOI:** 10.1097/MD.0000000000003175

**Published:** 2016-04-18

**Authors:** Eunyoung Lee, Hyung Jung Oh, Jung Tak Park, Seung Hyeok Han, Dong-Ryeol Ryu, Shin-Wook Kang, Tae-Hyun Yoo

**Affiliations:** From the Department of Internal Medicine, Yonsei University College of Medicine (EL, HJO, JTP, SHH, S-WK, THY), Department of Internal Medicine, School of Medicine, Ewha Womans University (D-RR), and Severance Biomedical Science Institute, Brain Korea 21 PLUS, Yonsei University (S-WK), Seoul, Republic of Korea.

## Abstract

Diabetic kidney disease leads to microalbuminuria and gradually progresses to overt proteinuria with renal insufficiency. Recent studies have demonstrated that 20% to 40% of patients with diabetic kidney disease are normo- to microalbuminuric, despite reduced renal function. We investigated renal and cardiovascular outcomes in patients with diabetes and renal insufficiency who were normo-, micro-, and macroalbuminuric.

Patients with diabetes and stage III or IV chronic kidney disease were recruited and divided into normoalbuminuric, microalbuminuric, and macroalbuminuric groups. New-onset cardiovascular events and renal outcomes, defined by end-stage renal disease or a 50% decline in estimated glomerular filtration rate, were evaluated.

Among the 1136 study patients, 255 (22.4%) were normoalbuminuric. During a mean follow-up duration of 44 months, the incidence of cardiovascular disease was similar among groups (*P* = 0.68). However, renal outcomes were significantly more common in patients with macroalbuminuria than in those who were normoalbuminuric or microalbuminuric (*P* < 0.001). Multivariate Cox analysis identified macroalbuminuria and estimated glomerular filtration rate as independent predictors of renal outcomes. The amount of albuminuria was not associated with cardiovascular events in this population.

Although cardiovascular events were similar in patients with diabetic kidney disease and renal insufficiency, renal outcomes differed significantly according to the amount of albuminuria.

## INTRODUCTION

The prevalence of diabetes mellitus has increased as a result of extended average life expectancy and an increasing rate of obesity. As the rate of diabetes has risen, diabetic kidney disease (DKD), a chronic microvascular complication, has also become more prevalent. DKD is characterized by albuminuria and reduced renal function, and is the leading cause of end-stage renal disease (ESRD), accounting for 42% of ESRD patients starting renal replacement therapy.^1^ Atherosclerotic cardiovascular disease (CVD) is also highly prevalent in patients with diabetes, especially in patients with reduced renal function. Moreover, DKD is significantly associated with high mortality and morbidity caused by CVD.^2,3^ Although the histologic findings and pathophysiologic mechanisms of DKD are relatively similar in patients with type 1 and 2 diabetes mellitus, certain differences exist in the natural course of DKD progression. Patients with type 1 diabetes develop microalbuminuria approximately 10 to 15 years after the onset of diabetes, and this condition subsequently progresses to overt proteinuria, consequent hypertension, and renal insufficiency.^4^ In patients with type 2 diabetes, more heterogeneous clinical presentations, including albuminuria, hypertension, and reduced renal function, are demonstrated, even at the onset of diabetes mellitus.^5^ Albuminuria, including microalbuminuria, is one of the earliest clinical signs of DKD and is a marker of renal progression and an increased risk of cardiovascular morbidity.^6^ Since early and aggressive treatment of albuminuria can alleviate or delay DKD progression,^7^ appropriate screening at an early stage is mandatory in these patients. According to a United Kingdom Prospective Diabetes Study report, patients with DKD and macroalbuminuria progress to renal failure, although a substantial number of patients with diabetes without albuminuria and those with microalbuminuria also show renal dysfunction.^8^ MacIsaac et al^9^ also reported that 39% of diabetic patients with advanced (over stage 3) chronic kidney disease (CKD) had urine albumin levels in the normal range. In the DEMAND study, a recent cross-sectional study, patients with normoalbuminuria and advanced renal insufficiency accounted for 20% of the total, and these patients also had a high risk of CVD.^10^ In Asian populations, patients with diabetes without albuminuria often show a decline in renal function, and the incidence of CVD is relatively similar between patients with macroalbuminuria and normoalbuminuria.^11^ These findings suggest that decreased renal function per se is an independent prognostic factor of cardiovascular outcome in patients with diabetes, irrespective of the amount of albuminuria. The Kidney Disease Outcomes Quality Initiative (KDOQI) guidelines recommend that patients with diabetes should be screened by simultaneously measuring the urinary albumin-to-creatinine ratio (ACR) and estimated glomerular filtration rate (eGFR).^12^ The KDOQI also mentioned special circumstances in which to suspect nondiabetic renal disease, such as in patients with diabetes and a low eGFR who do not have microalbuminuria or macroalbuminuria or other microvascular complications, including diabetic retinopathy. Based on previous studies, renal insufficiency accompanied by normoalbuminuria is relatively common in patients with diabetes but may reflect disease progression in terms of renal or cardiovascular outcome. However, few studies have investigated the long-term prognosis of these patients while focusing on renal and cardiovascular outcomes. The present study aimed to ascertain these clinical differences by comparing both the development of cardiovascular complications and the progression to ESRD or a 50% decline in eGFR in patients with diabetes and renal insufficiency according to the amount of albuminuria.

## METHODS

### Study Population

Between January 2007 and December 2009, 14,245 patients with type 2 diabetes mellitus visited our outpatient clinics. Demographic and laboratory data were retrospectively retrieved from the Yonsei Electronic Medical Record Database. The eGFR was calculated from serum creatinine levels using the Chronic Kidney Disease Epidemiology Collaboration equation.^13^ Patients with type 2 diabetes and impaired renal function (eGFR < 60 mL/min/1.73 m^2^) were included in this analysis. The amount of albuminuria was assessed by spot urine ACR. Study subjects were divided according to the amount of albuminuria into normoalbuminuria (ACR < 30 mg/g), microalbuminuria (ACR 30–300 mg/g), and macroalbuminuria (ACR > 300 mg/g) groups. Criteria for exclusion were extremely young or old age (<18 years or >75 years), acute kidney injury, and preserved renal function (eGFR > 60 mL/min/1.73 m^2^). Patients with stage 5 CKD or patients on dialysis therapy were also excluded. We also excluded patients for whom ACRs were not available and those with a relatively short follow-up duration (<6 months). This study was carried out in accordance with the Declaration of Helsinki and approved by the Institutional Review Board (IRB) of Yonsei University Health System Clinical Trial Center. Since this was a retrospective study and subjects were deidentified, the IRB waived the need to obtain written informed consent from the study subjects.

### Data Collection

Demographic and clinical data collected at the time of study enrollment included age, sex, systolic and diastolic blood pressure, history of CVD, and laboratory parameters, including blood urea nitrogen (BUN), creatinine, and lipid profiles including total cholesterol, triglyceride, HDL, and LDL cholesterol levels, and spot urine ACR.

### Study Endpoints

The cardiovascular composite endpoint was any major cardiovascular and cerebrovascular event. A major cardiovascular event was defined as cardiovascular mortality and hospitalization for acute coronary syndrome or coronary revascularization, including percutaneous coronary intervention or coronary artery bypass graft. Acute coronary syndrome was defined as presentation with chest pain and/or ischemic electrocardiographic changes and elevation of troponin T, with evidence of infarct by stress imaging or coronary angiography and ventriculography. Cerebrovascular events included both nonfatal ischemic stroke and nonfatal hemorrhagic stroke. Renal outcome was defined as a 50% decline in eGFR or development of ESRD, which was defined as initiation of renal replacement therapy, including hemodialysis, peritoneal dialysis, or kidney transplantation. In subgroup analysis, we defined good glycemic control group as hemoglobin A1c (HbA1c) below 8% and poor glycemic control group as HbA1c above 8%.^14^ All events were retrieved from the database and carefully reviewed to determine the incidence of cardiovascular outcomes or renal outcomes.

### Statistical Analysis

All variables are expressed as the mean ± standard deviation or percentage. Normality of the distribution of variables was tested by the Kolmogorov–Smirnov test. Comparisons were evaluated by Chi-square test, Student *t* test, or analysis of variance (ANOVA). Survival curves were created using the Kaplan–Meier method, and comparisons were made using the log-rank test. A Cox proportional hazards model was used to identify independent variables affecting event-free survival, and the results are presented as hazard ratio (HR) and 95% confidence interval (CI). Possible bias from different baseline characteristics among the 3 groups was calibrated by propensity score matching using logistic regression with the independent variables including age, duration of diabetes, eGFR, and any underlying CVD. Propensity score matching was conducted with greedy nearest neighbor matching using SAS version 9.1 (SAS Institute, Cary, NC).^15^ After all propensity score matches were performed, we assessed the balance in baseline covariates among the three groups using the ANOVA test for continuous variables and the Chi-square test for categorical variables. Results of time-dependent covariate analysis, which was used to confirm the assumption of proportionality, were not statistically significant, suggesting that the proportional hazards assumption is reasonable. All tests were 2 sided, and *P* < 0.05 was considered significant. Statistical analyses were performed with SPSS for Windows, version 18.0 (SPSS, Inc., Chicago, IL).

## RESULTS

### Baseline Patient Characteristics According to the Amount of Albuminuria

**FIGURE 1:**
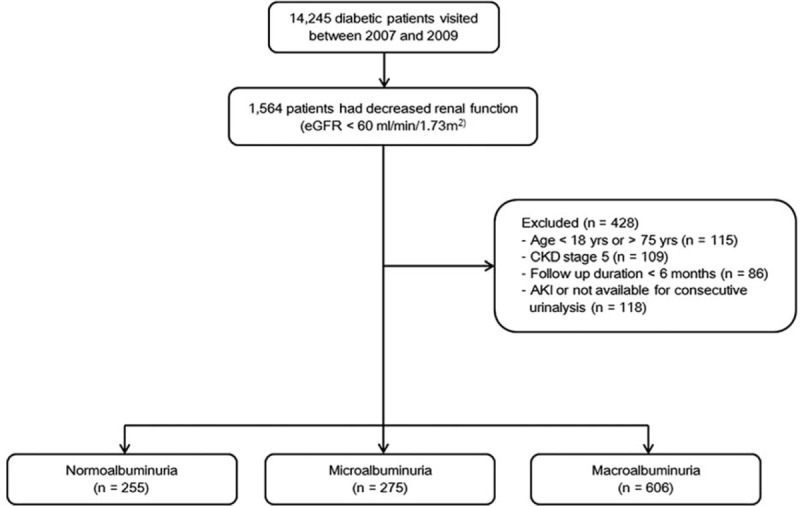
Flow chart of the study population. Patients with diabetes who visited our clinic between 2007 and 2009 were initially screened for enrollment. A total of 1136 patients with diabetes and stage III or IV chronic kidney disease (CKD) were analyzed.

Among the 14,245 patients with type 2 diabetes initially screened, 1564 had decreased renal function (eGFR < 60 mL/min/1.73 m^2^). After 428 patients were excluded, the final analysis included 1136 patients who were then classified into 3 groups according to the amount of albuminuria: a normoalbuminuria group (255 patients, 22.4%), a microalbuminuria group (275 patients, 24.2%), and a macroalbuminuria group (606 patients, 53.3%) (Figure 1). The mean age was 61.7 years, and 391 patients (34.4%) were women. The mean eGFR was 38.9 ± 13.9 mL/min/1.73 m^2^. The mean age and eGFR were significantly lower in the macroalbuminuria group, whereas creatinine and HbA1c levels were significantly higher in the macroalbuminuria group than in the normoalbuminuria and microalbuminuria groups. Patients in the macroalbuminuria group had a longer duration of diabetes, whereas a history of CVD was less prevalent in the macroalbuminuria group than in the normoalbuminuria or microalbuminuria groups. No significant differences in sex, body mass index (BMI), hypertension or lipid profiles were found among the 3 groups (Table 1).

**TABLE 1:**
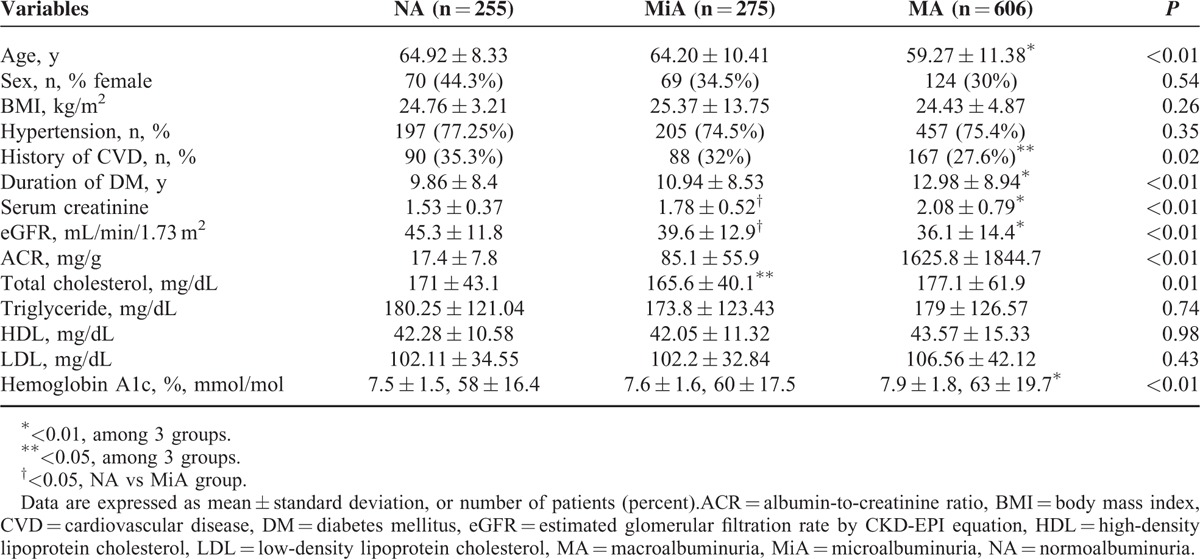
Baseline Characteristics of Study Subjects According to the Amount of Albuminuria

### Cardiovascular and Renal Outcomes

During a mean follow-up duration of 44 months, new-onset cardiovascular events developed in 191 patients (16.8%). These events included 127 cardiovascular events and 50 cerebrovascular events. There were 14 patients who had both cardiovascular and cerebrovascular events. The incidence of cardiovascular events was 3.90 per 1000 person-years in the normoalbuminuria group, 4.21 per 1000 person-years in the microalbuminuria group, and 4.10 per 1000 person-years in the macroalbuminuria group. Cardiovascular outcomes were comparable among the three groups. However, renal outcomes, defined by progression to ESRD or a 50% decline in eGFR, were significantly higher in the macroalbuminuria group than in the normoalbuminuria and microalbuminuria groups (9.88, 0.63, and 0.95 per 1000 person-years in the macroalbuminuria, normoalbuminuria, and microalbuminuria groups, respectively; *P* < 0.01; Table 2).

**TABLE 2:**
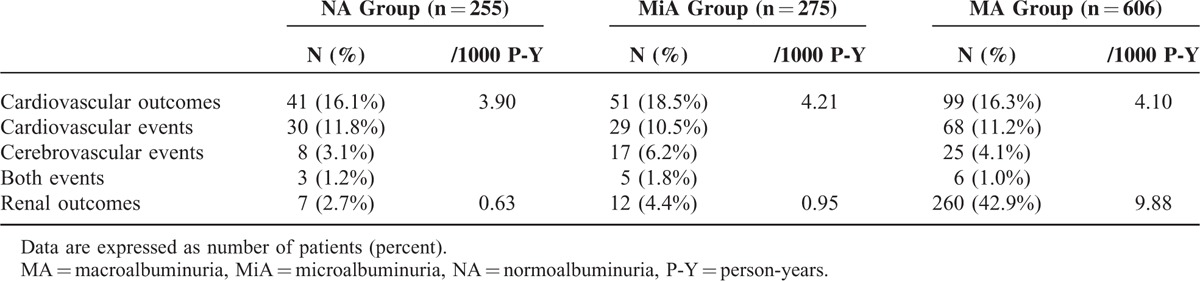
Incidence of Cardiovascular Events and Renal Outcomes According to the Amount of Albuminuria

**FIGURE 2:**
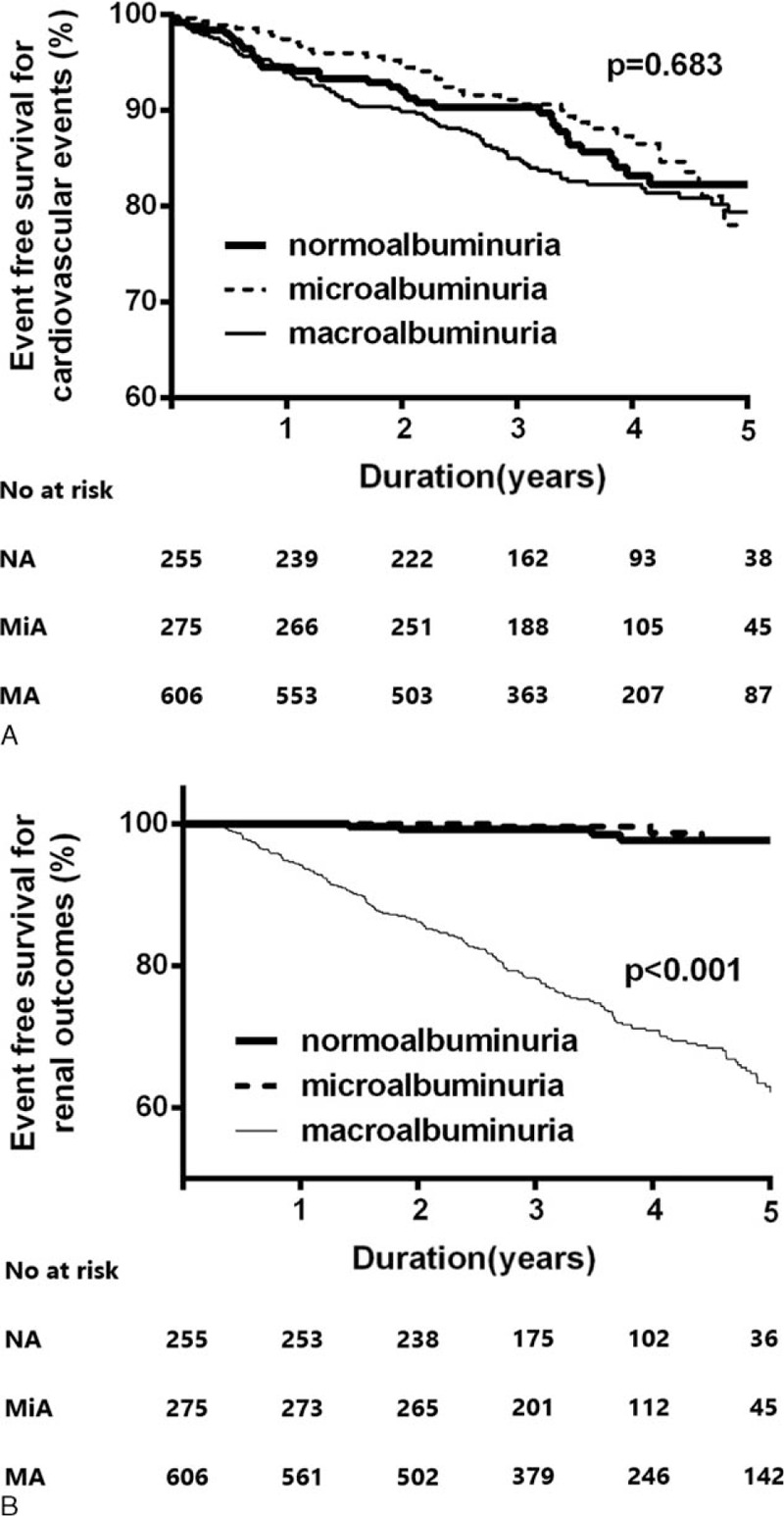
Kaplan–Meier plots for cardiovascular outcomes (A) and renal outcomes (B) according to the amount of albuminuria. New-onset cardiovascular events are comparable among the three groups (*P* = 0.68). However, macroalbuminuric patients have a significantly higher risk of renal outcomes (defined by a 50% decline in eGFR or ESRD) compared with patients with normoalbuminuria and microalbuminuria (*P* < 0.001).

Kaplan–Meier plots revealed no significant differences in new-onset cardiovascular events among the three groups (*P* = 0.68; Figure 2A). However, in terms of renal outcomes, patients in the macroalbuminuria group had a significantly higher risk of a 50% decline of eGFR or ESRD compared with those in the normoalbuminuria and microalbuminuria groups (*P* < 0.001; Figure 2B). We performed a Cox proportional hazard analysis to identify independently associated factors. The amount of albuminuria in study subjects was not a significant risk factor for cardiovascular events (HR, 1.138; 95% CI: 0.941–1.376; *P* = 0.18) in the study population. CVD history and hypertension, however, were independent risk factors for new-onset cardiovascular events (HR, 1.947; 95% CI: 1.441–2.631; *P* < 0.01 and HR, 1.384; 95% CI: 1.010–1.967; *P* = 0.04, respectively; Table 3). Meanwhile, after adjusting for confounding variables, the amount of albuminuria (HR: 2.472, 95% CI: 1.988–3.075, *P* < 0.01) and eGFR (HR: 0.970, 95% CI: 0.959–0.981, *P* < 0.01) were independent risk factors for renal outcomes.

**TABLE 3:**
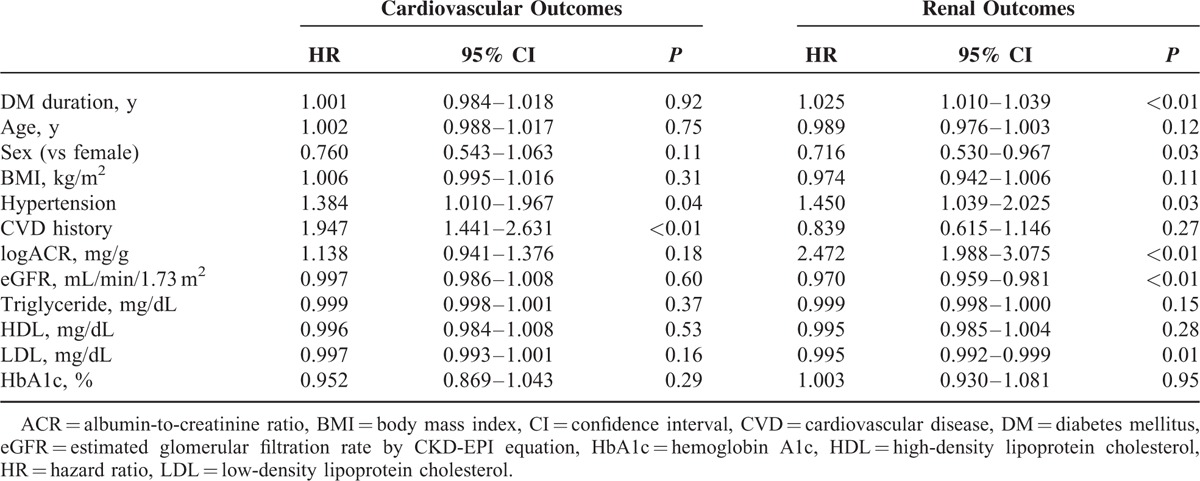
Multivariate Cox Proportional Hazard Analysis for Cardiovascular and Renal Outcomes

**FIGURE 3:**
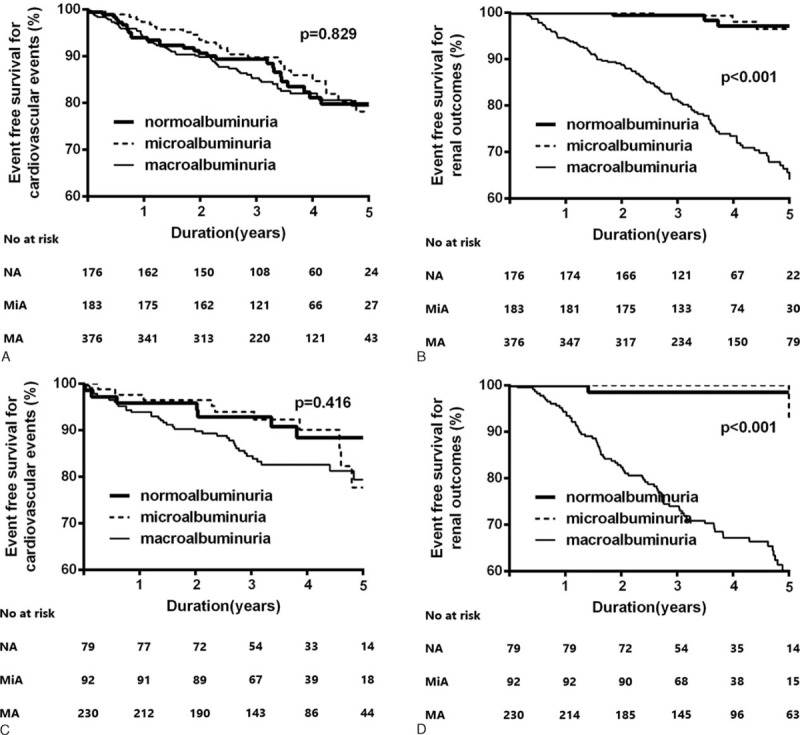
Kaplan–Meier plots for cardiovascular outcomes (A) and renal outcomes (B) according to the amount of albuminuria in patients with good glycemic control (≤8%) and cardiovascular outcomes (C) and renal outcomes (D) according to the amount of albuminuria in patients with poor glycemic control (>8%). Cardiovascular outcomes are not different among the three groups in both good glycemic control group (*P* = 0.83) and poor glycemic control group (*P* = 0.42). The renal outcomes in the macroalbuminuria group are significantly higher compared with those of the normo- and microalbuminuria groups in both good glycemic control group (*P* < 0.001) and poor glycemic control group (*P* < 0.001).

In addition, subgroup analysis was conducted in good and poor glycemic control group. CVD history was an independent risk factor of cardiovascular outcomes in both subgroups. For renal outcomes, the amount of albuminuria and eGFR were independent risk factor in both subgroups (Table 4). Kaplan–Meier survival analysis also showed same trends among the 3 groups according to level of albuminuria even in subgroup according to good or poor glycemic control (Figure 3).

**TABLE 4:**
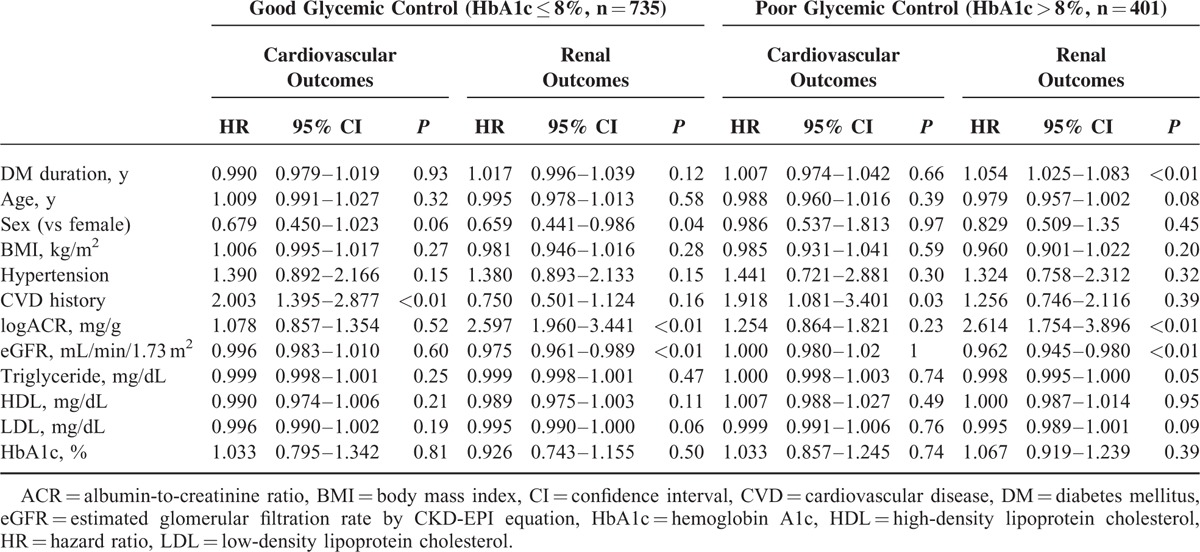
Multivariate Cox Proportional Hazard Analysis for Cardiovascular and Renal Outcomes According to Glycemic Control

### Propensity Score Matching Analysis

**FIGURE 4:**
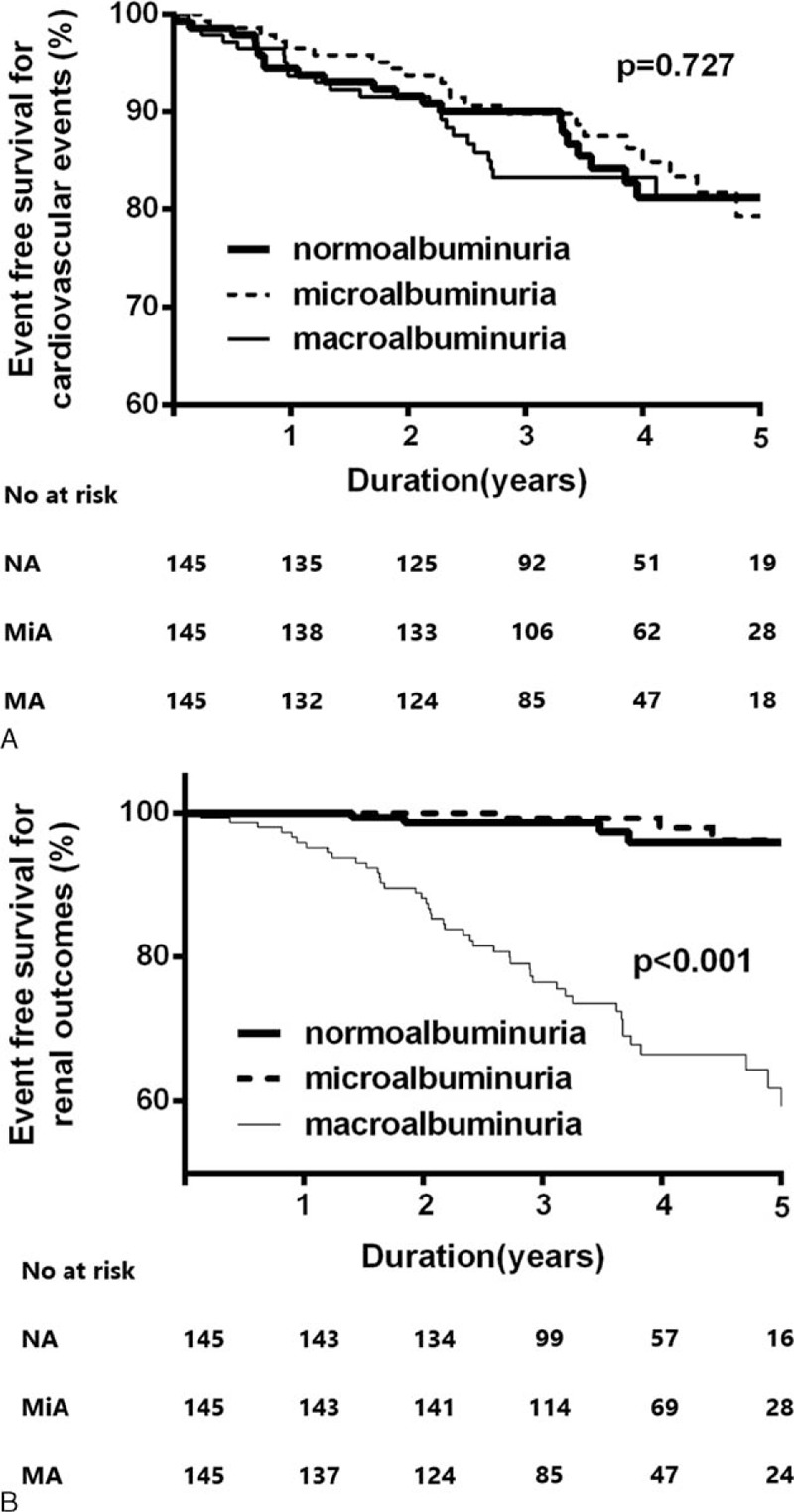
Kaplan–Meier plots for cardiovascular outcomes (A) and renal outcomes (B) according to the amount of albuminuria after propensity score matching. Cardiovascular outcomes are not different among the three groups (*P* = 0.73). The renal outcomes in the macroalbuminuria group are significantly higher compared with those of the normo- and microalbuminuria groups (*P* < 0.001).

To adjust for differences in baseline characteristics, we performed propensity score matching using four variables (age, eGFR, duration of diabetes, and history of CVD), selecting 145 patients in each group (Table 5). Kaplan–Meier plots also showed that cardiovascular outcome did not differ among the normoalbuminuria, microalbuminuria, and macroalbuminuria groups (*P* = 0.73; Figure 4A), although patients in the macroalbuminuria group still had a significantly higher risk of renal progression than patients in the normoalbuminuric or microalbuminuric groups (*P* < 0.001; Figure 4B).

**TABLE 5:**
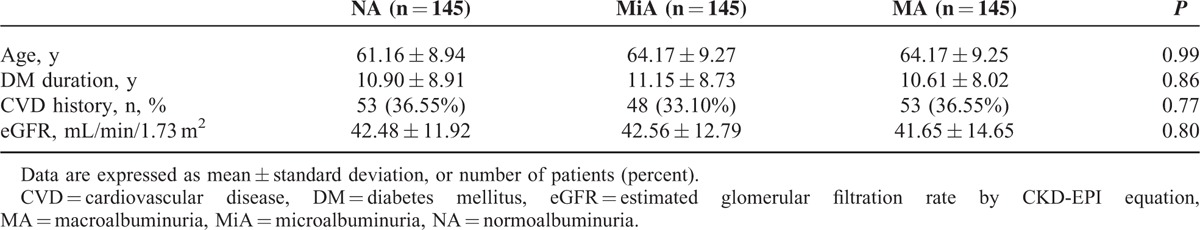
Demographic Characteristics of Subjects After Propensity Score Matching

## DISCUSSION AND CONCLUSIONS

This study was performed to investigate the clinical characteristics of patients with diabetes and decreased renal function by comparing cardiovascular and renal outcomes according to the amount of albuminuria. We demonstrated that cardiovascular outcomes were comparable among the normoalbuminuric, microalbuminuric, and macroalbuminuric groups of patients with DKD and reduced renal function. Meanwhile, the amount of albuminuria was closely related to renal outcomes in patients with DKD and renal insufficiency, as previously reported.^16^

Chronic diabetic complications are classified as microvascular or macrovascular according to the size of the affected vessel and affected organs. Diabetic nephropathy, as a representative microvascular complication of diabetes, is characterized by albuminuria and subsequent renal dysfunction.^17^ Albuminuria is a distinctive sign of diabetic nephropathy and is also closely associated with the development of cardiovascular events.^18,19^ Macrovascular complications are defined as premature accelerated atherosclerosis in medium to large vessels, and its related signs and symptoms vary depending on the affected artery or organ.^20,21^ eGFR reflects renal function and is influenced by the number of functional nephrons or the amount of renal blood flow. Glomerular diseases, including DKD, are associated with albuminuria, consequent capillary damage, and glomerulosclerosis.^22^ Diabetic glomerulosclerosis is associated with a decreased surface area of the glomerular capillary network and a decline in eGFR.^23^ In addition, chronic renal hypoperfusion related to atherosclerosis of the renal artery reduces the glomerular capillary ultrafiltration coefficient and efferent arteriolar blood flow and can also induce global sclerosis, ultimately causing a progressive decrease in eGFR.^24^ Hemodynamic changes due to atherosclerotic renal disease as a chronic macrovascular complication might be associated with isolated renal dysfunction without albuminuria in patients with diabetes. In addition, macrovascular disease is frequently accompanied by profound atherosclerosis in another area, such as the cardiovascular or cerebrovascular system.^21,22^ A previous study also demonstrated that patients with normoalbuminuric type 2 diabetes with reduced eGFR presented with a pattern of intrarenal vascular disease similar to those with micro- and macroalbuminuria with renal impairment.^9,25^ A previous cross-sectional study showed that patients with normoalbuminuric diabetic CKD were more likely to have a history of CVD.^26^ Patients with normoalbuminuric advanced CKD also had a higher incidence of macroangiopathies than patients with normoalbuminuric stage 1 or 2 CKD in that study. The present study also revealed that the incidence of cardiovascular events was similar between patients with normoalbuminuric diabetes with renal dysfunction and patients with macroalbuminuric diabetic CKD. Based on the results of this study and previous studies, it appears that isolated renal dysfunction without overt albuminuria reflects a macrovascular complication and is associated with a relatively high incidence of CVD events in patients with DKD. As expected, the present study showed that an isolated decline in eGFR with insignificant albuminuria was not associated with poor renal outcome, despite a similar incidence of cardiovascular events among patients with DKD. Meanwhile, there was a significant association between the amount of albuminuria and the progression to a 50% decline in eGFR or ESRD in this study population.

An increasing number of studies on diabetic patients with renal insufficiency without albuminuria have published in recent years.^27,28^ Several studies described clinical differences between patients with macroalbuminuric and normoalbuminuric diabetes^10,26,29^ but few studies have investigated the long-term prognosis in this population. We classified patients into 3 groups (normoalbuminuria, microalbuminuria, and macroalbuminuria) based on the amount of albuminuria. In our study, 22.4% of patients with DKD were normoalbuminuric, which is in line with previous findings.^10,27^ Patients in the macroalbuminuric group were much younger and had a longer duration of diabetes compared with those in the normoalbuminuria and microalbuminuria groups. Generally, albuminuria as a chronic diabetic microvascular complication is a marker of glomerular capillary damage and endothelial dysfunction, and is also associated with a longer exposure to hyperglycemia in diabetes.^30^ Rigalleau et al^31^ also reported that normoalbuminuric CKD patients had a lower duration of diabetes and a lower incidence of diabetic retinopathy. In contrast, the prevalence of hypertension was similar among patients with normo-, micro-, and macroalbuminuria with a reduced eGFR. MacIsaac et al^9^ reported that patients with normoalbuminuria were significantly older, which is consistent with our observation. Taken together, the older age and shorter duration of diabetes in the normoalbuminuria group suggest that isolated decreased eGFR in patients with normoalbuminuria is associated with atherosclerotic vascular damage, such as nephrosclerosis or arterial hyalinosis, rather than chronic diabetic glomerular injury.^32^

Because of the distinct baseline characteristics of patients in our study population, some important baseline covariates, including history of CVD and age, were not distributed equally. Patients in the normoalbuminuria group were significantly older, had a shorter duration of diabetes, and had relatively preserved renal function, compared with those in the other groups. Moreover, the development of cardiovascular complications might be influenced by a patient's history of CVD and other risk factors, such as age. Therefore, we used propensity score matching and created among the 3 groups an identical matched cohort at baseline. In this matched cohort, comparable cardiovascular outcomes and distinguishable renal outcomes were also observed among patients with normo-, micro-, and macroalbuminuric diabetic CKD.

This study has several potential limitations that should be noted. Because it was an observational retrospective study, possible bias could not be excluded. In addition, because subjects were screened at a tertiary hospital outpatient clinic of a single center, certain characteristics of patients who presented to the hospital might have caused a selection bias (e.g., a history of CVD and or more severe comorbid conditions). Second, single measurements of the ACR were used for analysis. Since the albumin excretion rate might be variable in special circumstances (e.g., dehydration, exercise), the absence of serial follow-up measurements was another limitation of the present study. To compensate for this limitation, we additionally performed an R square analysis from some of study subjects, who are available the ACR values 3-month after study enrollment. A total of 698 values of ACR at third month were obtained. The adjusted R square of the initial ACR and the 3-month ACR was 0.94, which is significantly correlated. It seems to be closely correlated with initial and third month ACR. Nevertheless, we acknowledge this is limitation of our study. Finally, we did not include medication history (e.g., angiotensin-converting enzyme inhibitors or angiotensin receptor blockers), and it is possible that the use of such medications affects the amount of albuminuria. The use of statins or antiplatelet agents might also affect cardiovascular outcomes. Therefore, the lack of data regarding concomitant medication history is another limitation of this study. Despite these limitations, we conclude that, irrespective of the amount of albuminuria, patients with diabetic CKD have a higher incidence of CVD and isolated renal dysfunction without significant albuminuria, and should be considered as being at high risk for CVD.

In conclusion, there are significant and substantial differences among diabetic patients with renal dysfunction according to their level of albuminuria. However, the incidence of cardiovascular events among normoalbuminuric, microalbuminuric, and macroalbuminuric groups is comparable in patients with diabetes and advanced renal insufficiency.
